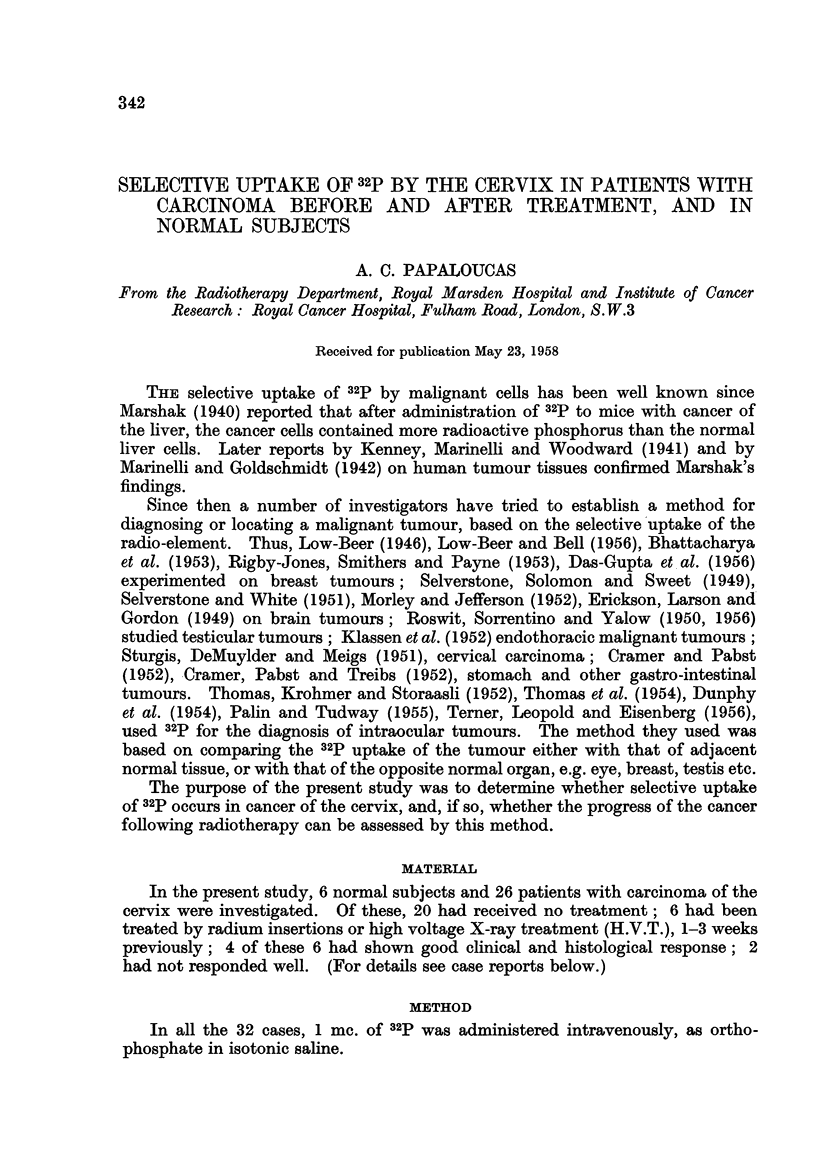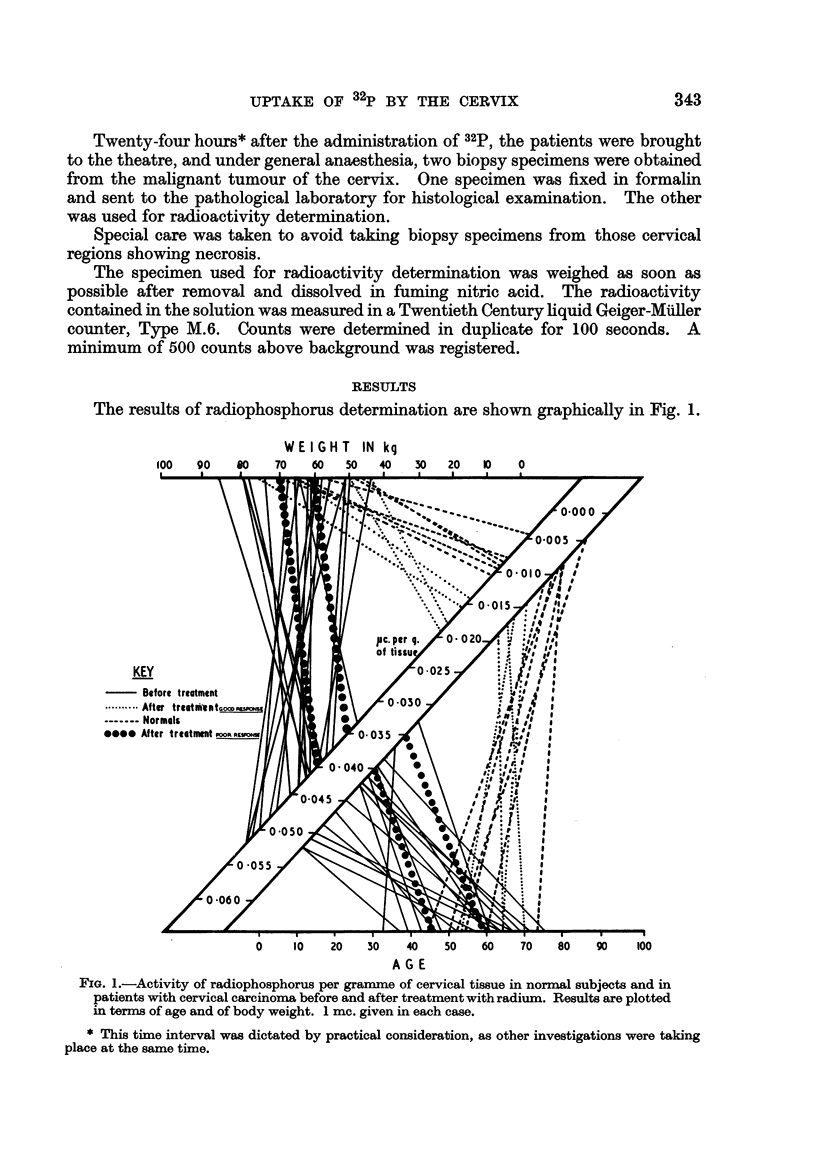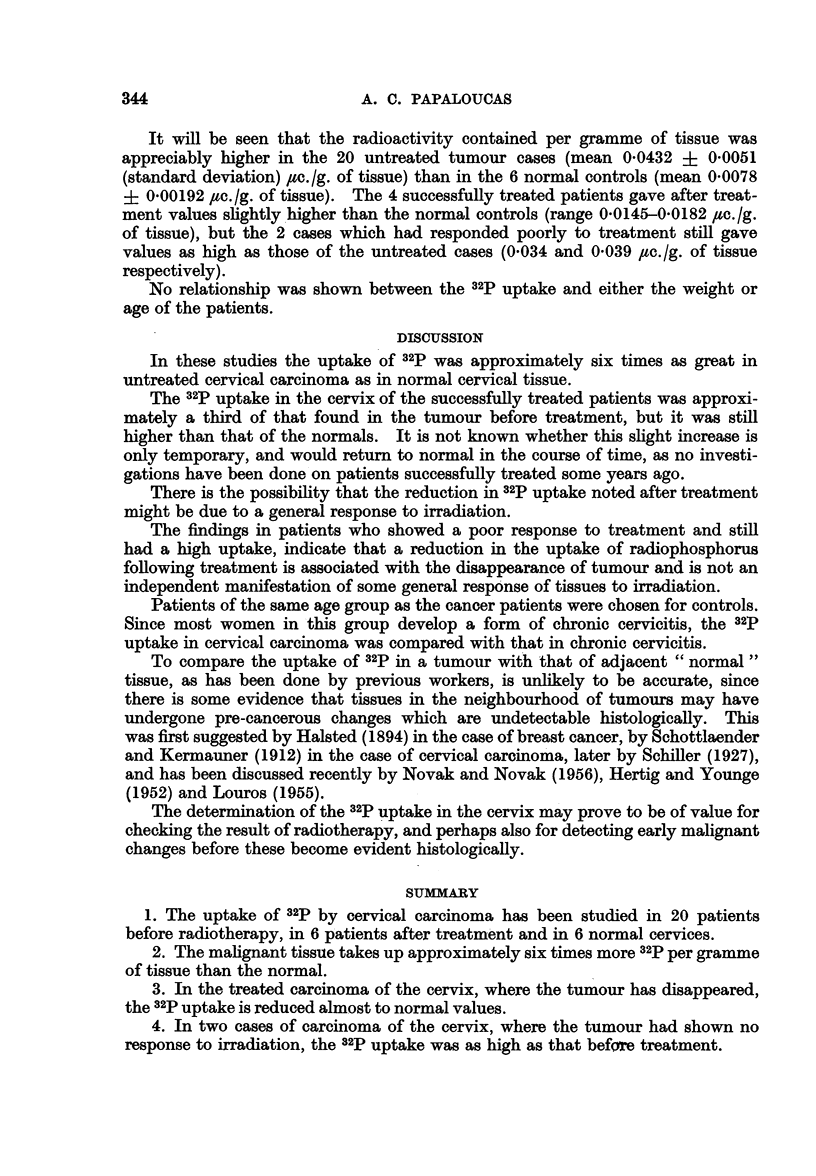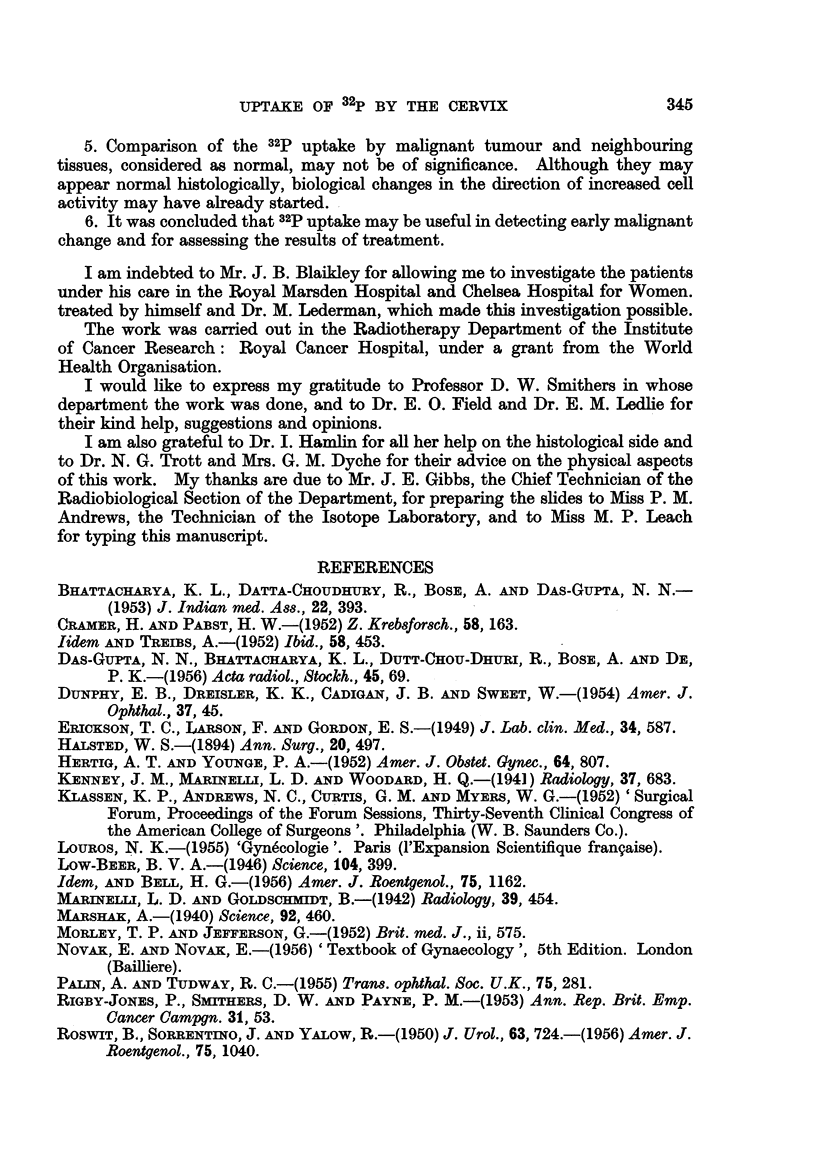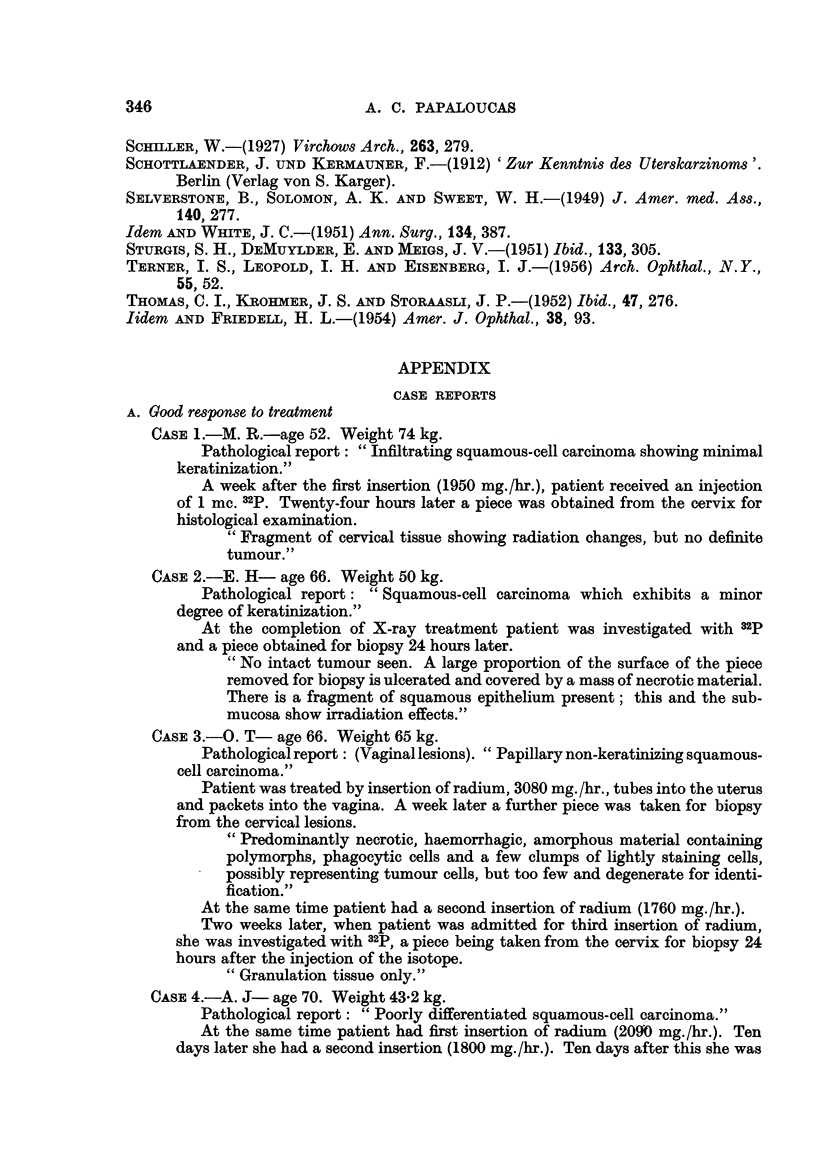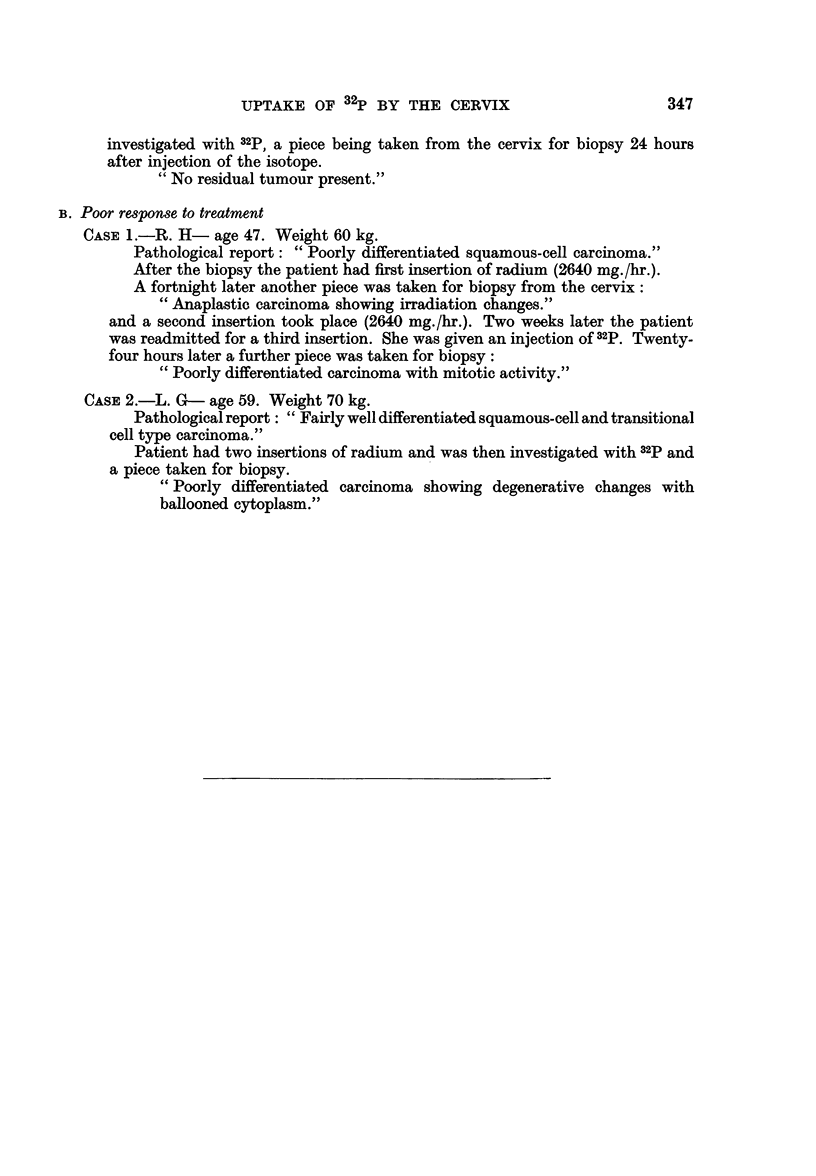# Selective Uptake of ^32^P by the Cervix in Patients with Carcinoma before and after Treatment, and in Normal Subjects

**DOI:** 10.1038/bjc.1958.41

**Published:** 1958-09

**Authors:** A. C. Papaloucas


### SELECTIVE UPTAKE OF $^{32}P$ BY THE CERVIX IN PATIENTS WITH CARCINOMA BEFORE AND AFTER TREATMENT, AND IN NORMAL SUBJECTS

A. C. PAPALOUCAS

*From the Radiotherapy Department, Royal Marsden Hospital and Institute of Cancer Research : Royal Cancer Hospital, Fulham Road, London, S.W.3*

Received for publication May 23, 1958

THE selective uptake of $^{32}P$ by malignant cells has been well known since Marshak (1940) reported that after administration of $^{32}P$ to mice with cancer of the liver, the cancer cells contained more radioactive phosphorus than the normal liver cells. Later reports by Kenney, Marinelli and Woodward (1941) and by Marinelli and Goldschmidt (1942) on human tumour tissues confirmed Marshak's findings.

Since then a number of investigators have tried to establish a method for diagnosing or locating a malignant tumour, based on the selective uptake of the radio-element. Thus, Low-Beer (1946), Low-Beer and Bell (1956), Bhattacharya *et al.* (1953), Rigby-Jones, Smithers and Payne (1953), Das-Gupta *et al.* (1956) experimented on breast tumours ; Selverstone, Solomon and Sweet (1949), Selverstone and White (1951), Morley and Jefferson (1952), Erickson, Larson and Gordon (1949) on brain tumours ; Roswit, Sorrentino and Yalow (1950, 1956) studied testicular tumours ; Klassen *et al.* (1952) endothoracic malignant tumours ; Sturgis, DeMuylder and Meigs (1951), cervical carcinoma ; Cramer and Pabst (1952), Cramer, Pabst and Treibs (1952), stomach and other gastro-intestinal tumours. Thomas, Krohmer and Storaasli (1952), Thomas *et al.* (1954), Dunphy *et al.* (1954), Palin and Tudway (1955), Terner, Leopold and Eisenberg (1956), used $^{32}P$ for the diagnosis of intraocular tumours. The method they used was based on comparing the $^{32}P$ uptake of the tumour either with that of adjacent normal tissue, or with that of the opposite normal organ, e.g. eye, breast, testis etc.

The purpose of the present study was to determine whether selective uptake of $^{32}P$ occurs in cancer of the cervix, and, if so, whether the progress of the cancer following radiotherapy can be assessed by this method.

### MATERIAL

In the present study, 6 normal subjects and 26 patients with carcinoma of the cervix were investigated. Of these, 20 had received no treatment ; 6 had been treated by radium insertions or high voltage X-ray treatment (H.V.T.), 1–3 weeks previously ; 4 of these 6 had shown good clinical and histological response ; 2 had not responded well. (For details see case reports below.)

### METHOD

In all the 32 cases, 1 mc. of $^{32}P$ was administered intravenously, as orthophosphate in isotonic saline.

Twenty-four hours* after the administration of $^{32}$P, the patients were brought to the theatre, and under general anaesthesia, two biopsy specimens were obtained from the malignant tumour of the cervix. One specimen was fixed in formalin and sent to the pathological laboratory for histological examination. The other was used for radioactivity determination.

Special care was taken to avoid taking biopsy specimens from those cervical regions showing necrosis.

The specimen used for radioactivity determination was weighed as soon as possible after removal and dissolved in fuming nitric acid. The radioactivity contained in the solution was measured in a Twentieth Century liquid Geiger-Müller counter, Type M.6. Counts were determined in duplicate for 100 seconds. A minimum of 500 counts above background was registered.

### RESULTS

The results of radiophosphorus determination are shown graphically in Fig. 1.

FIG. 1.—Activity of radiophosphorus per gramme of cervical tissue in normal subjects and in patients with cervical carcinoma before and after treatment with radium. Results are plotted in terms of age and of body weight. 1 mc. given in each case.

* This time interval was dictated by practical consideration, as other investigations were taking place at the same time.

It will be seen that the radioactivity contained per gramme of tissue was appreciably higher in the 20 untreated tumour cases (mean 0·0432 $\pm$ 0·0051 (standard deviation) $\mu$c./g. of tissue) than in the 6 normal controls (mean 0·0078 $\pm$ 0·00192 $\mu$c./g. of tissue). The 4 successfully treated patients gave after treatment values slightly higher than the normal controls (range 0·0145–0·0182 $\mu$c./g. of tissue), but the 2 cases which had responded poorly to treatment still gave values as high as those of the untreated cases (0·034 and 0·039 $\mu$c./g. of tissue respectively).

No relationship was shown between the $^{32}$P uptake and either the weight or age of the patients.

### DISCUSSION

In these studies the uptake of $^{32}$P was approximately six times as great in untreated cervical carcinoma as in normal cervical tissue.

The $^{32}$P uptake in the cervix of the successfully treated patients was approximately a third of that found in the tumour before treatment, but it was still higher than that of the normals. It is not known whether this slight increase is only temporary, and would return to normal in the course of time, as no investigations have been done on patients successfully treated some years ago.

There is the possibility that the reduction in $^{32}$P uptake noted after treatment might be due to a general response to irradiation.

The findings in patients who showed a poor response to treatment and still had a high uptake, indicate that a reduction in the uptake of radiophosphorus following treatment is associated with the disappearance of tumour and is not an independent manifestation of some general response of tissues to irradiation.

Patients of the same age group as the cancer patients were chosen for controls. Since most women in this group develop a form of chronic cervicitis, the $^{32}$P uptake in cervical carcinoma was compared with that in chronic cervicitis.

To compare the uptake of $^{32}$P in a tumour with that of adjacent " normal " tissue, as has been done by previous workers, is unlikely to be accurate, since there is some evidence that tissues in the neighbourhood of tumours may have undergone pre-cancerous changes which are undetectable histologically. This was first suggested by Halsted (1894) in the case of breast cancer, by Schottlaender and Kermauner (1912) in the case of cervical carcinoma, later by Schiller (1927), and has been discussed recently by Novak and Novak (1956), Hertig and Younge (1952) and Louros (1955).

The determination of the $^{32}$P uptake in the cervix may prove to be of value for checking the result of radiotherapy, and perhaps also for detecting early malignant changes before these become evident histologically.

### SUMMARY

1. The uptake of $^{32}$P by cervical carcinoma has been studied in 20 patients before radiotherapy, in 6 patients after treatment and in 6 normal cervices.

2. The malignant tissue takes up approximately six times more $^{32}$P per gramme of tissue than the normal.

3. In the treated carcinoma of the cervix, where the tumour has disappeared, the $^{32}$P uptake is reduced almost to normal values.

4. In two cases of carcinoma of the cervix, where the tumour had shown no response to irradiation, the $^{32}$P uptake was as high as that before treatment.

5. Comparison of the $^{32}P$ uptake by malignant tumour and neighbouring tissues, considered as normal, may not be of significance. Although they may appear normal histologically, biological changes in the direction of increased cell activity may have already started.

6. It was concluded that $^{32}P$ uptake may be useful in detecting early malignant change and for assessing the results of treatment.

I am indebted to Mr. J. B. Blaikley for allowing me to investigate the patients under his care in the Royal Marsden Hospital and Chelsea Hospital for Women. treated by himself and Dr. M. Lederman, which made this investigation possible.

The work was carried out in the Radiotherapy Department of the Institute of Cancer Research: Royal Cancer Hospital, under a grant from the World Health Organisation.

I would like to express my gratitude to Professor D. W. Smithers in whose department the work was done, and to Dr. E. O. Field and Dr. E. M. Ledlie for their kind help, suggestions and opinions.

I am also grateful to Dr. I. Hamlin for all her help on the histological side and to Dr. N. G. Trott and Mrs. G. M. Dyche for their advice on the physical aspects of this work. My thanks are due to Mr. J. E. Gibbs, the Chief Technician of the Radiobiological Section of the Department, for preparing the slides to Miss P. M. Andrews, the Technician of the Isotope Laboratory, and to Miss M. P. Leach for typing this manuscript.

### REFERENCES

BHATTACHARYA, K. L., DATTA-CHOUDHURY, R., BOSE, A. AND DAS-GUPTA, N. N.—(1953) *J. Indian med. Ass.*, **22**, 393.

CRAMER, H. AND PABST, H. W.—(1952) *Z. Krebsforsch.*, **58**, 163.

*Iidem* AND TREIBS, A.—(1952) *Ibid.*, **58**, 453.

DAS-GUPTA, N. N., BHATTACHARYA, K. L., DUTT-CHOU-DHURI, R., BOSE, A. AND DE, P. K.—(1956) *Acta radiol., Stockh.*, **45**, 69.

DUNPHY, E. B., DREISLER, K. K., CADIGAN, J. B. AND SWEET, W.—(1954) *Amer. J. Ophthal.*, **37**, 45.

ERICKSON, T. C., LARSON, F. AND GORDON, E. S.—(1949) *J. Lab. clin. Med.*, **34**, 587.

HALSTED, W. S.—(1894) *Ann. Surg.*, **20**, 497.

HERTIG, A. T. AND YOUNGE, P. A.—(1952) *Amer. J. Obstet. Gynec.*, **64**, 807.

KENNEY, J. M., MARINELLI, L. D. AND WOODARD, H. Q.—(1941) *Radiology*, **37**, 683.

KLASSEN, K. P., ANDREWS, N. C., CURTIS, G. M. AND MYERS, W. G.—(1952) ' Surgical Forum, Proceedings of the Forum Sessions, Thirty-Seventh Clinical Congress of the American College of Surgeons '. Philadelphia (W. B. Saunders Co.).

LOUROS, N. K.—(1955) 'Gynécologie '. Paris (l'Expansion Scientifique française).

LOW-BEER, B. V. A.—(1946) *Science*, **104**, 399.

*Idem*, AND BELL, H. G.—(1956) *Amer. J. Roentgenol.*, **75**, 1162.

MARINELLI, L. D. AND GOLDSCHMIDT, B.—(1942) *Radiology*, **39**, 454.

MARSHAK, A.—(1940) *Science*, **92**, 460.

MORLEY, T. P. AND JEFFERSON, G.—(1952) *Brit. med. J.*, ii, 575.

NOVAK, E. AND NOVAK, E.—(1956) ' Textbook of Gynaecology ', 5th Edition. London (Bailliere).

PALIN, A. AND TUDWAY, R. C.—(1955) *Trans. ophthal. Soc. U.K.*, **75**, 281.

RIGBY-JONES, P., SMITHERS, D. W. AND PAYNE, P. M.—(1953) *Ann. Rep. Brit. Emp. Cancer Campgn.* **31**, 53.

ROSWIT, B., SORRENTINO, J. AND YALOW, R.—(1950) *J. Urol.*, **63**, 724.—(1956) *Amer. J. Roentgenol.*, **75**, 1040.

SCHILLER, W.—(1927) *Virchows Arch.*, **263**, 279.
SCHOTTLAENDER, J. UND KERMAUNER, F.—(1912) '*Zur Kenntnis des Uterskarzinoms*'. Berlin (Verlag von S. Karger).
SELVERSTONE, B., SOLOMON, A. K. AND SWEET, W. H.—(1949) *J. Amer. med. Ass.*, **140**, 277.
*Idem* AND WHITE, J. C.—(1951) *Ann. Surg.*, **134**, 387.
STURGIS, S. H., DEMUYLDER, E. AND MEIGS, J. V.—(1951) *Ibid.*, **133**, 305.
TERNER, I. S., LEOPOLD, I. H. AND EISENBERG, I. J.—(1956) *Arch. Ophthal.*, *N.Y.*, **55**, 52.
THOMAS, C. I., KROHMER, J. S. AND STORAASLI, J. P.—(1952) *Ibid.*, **47**, 276.
*Iidem* AND FRIEDELL, H. L.—(1954) *Amer. J. Ophthal.*, **38**, 93.

### APPENDIX

### CASE REPORTS

A. *Good response to treatment*

CASE 1.—M. R.—age 52. Weight 74 kg.

Pathological report : " Infiltrating squamous-cell carcinoma showing minimal keratinization."

A week after the first insertion (1950 mg./hr.), patient received an injection of 1 mc. $^{32}$P. Twenty-four hours later a piece was obtained from the cervix for histological examination.

" Fragment of cervical tissue showing radiation changes, but no definite tumour."

CASE 2.—E. H— age 66. Weight 50 kg.

Pathological report : " Squamous-cell carcinoma which exhibits a minor degree of keratinization."

At the completion of X-ray treatment patient was investigated with $^{32}$P and a piece obtained for biopsy 24 hours later.

" No intact tumour seen. A large proportion of the surface of the piece removed for biopsy is ulcerated and covered by a mass of necrotic material. There is a fragment of squamous epithelium present ; this and the submucosa show irradiation effects."

CASE 3.—O. T— age 66. Weight 65 kg.

Pathological report : (Vaginal lesions). " Papillary non-keratinizing squamous-cell carcinoma."

Patient was treated by insertion of radium, 3080 mg./hr., tubes into the uterus and packets into the vagina. A week later a further piece was taken for biopsy from the cervical lesions.

" Predominantly necrotic, haemorrhagic, amorphous material containing polymorphs, phagocytic cells and a few clumps of lightly staining cells, possibly representing tumour cells, but too few and degenerate for identification."

At the same time patient had a second insertion of radium (1760 mg./hr.).

Two weeks later, when patient was admitted for third insertion of radium, she was investigated with $^{32}$P, a piece being taken from the cervix for biopsy 24 hours after the injection of the isotope.

" Granulation tissue only."

CASE 4.—A. J— age 70. Weight 43·2 kg.

Pathological report : " Poorly differentiated squamous-cell carcinoma."

At the same time patient had first insertion of radium (2090 mg./hr.). Ten days later she had a second insertion (1800 mg./hr.). Ten days after this she was investigated with $^{32}$P, a piece being taken from the cervix for biopsy 24 hours after injection of the isotope.

"No residual tumour present."

B. *Poor response to treatment*

CASE 1.—R. H— age 47. Weight 60 kg.

Pathological report: "Poorly differentiated squamous-cell carcinoma."

After the biopsy the patient had first insertion of radium (2640 mg./hr.).

A fortnight later another piece was taken for biopsy from the cervix:

"Anaplastic carcinoma showing irradiation changes."

and a second insertion took place (2640 mg./hr.). Two weeks later the patient was readmitted for a third insertion. She was given an injection of $^{32}$P. Twenty-four hours later a further piece was taken for biopsy:

"Poorly differentiated carcinoma with mitotic activity."

CASE 2.—L. G— age 59. Weight 70 kg.

Pathological report: "Fairly well differentiated squamous-cell and transitional cell type carcinoma."

Patient had two insertions of radium and was then investigated with $^{32}$P and a piece taken for biopsy.

"Poorly differentiated carcinoma showing degenerative changes with ballooned cytoplasm."